# Epigenetic and transgenerational mechanisms in infection-mediated neurodevelopmental disorders

**DOI:** 10.1038/tp.2017.78

**Published:** 2017-05-02

**Authors:** U Weber-Stadlbauer

**Affiliations:** 1 Institute of Pharmacology and Toxicology, University of Zurich-Vetsuisse, Zurich, Switzerland

## Abstract

Prenatal infection is an environmental risk factor for various brain disorders with neurodevelopmental components, including autism spectrum disorder and schizophrenia. Modeling this association in animals shows that maternal immune activation negatively affects fetal brain development and leads to the emergence of behavioral disturbances later in life. Recent discoveries in these preclinical models suggest that epigenetic modifications may be a critical molecular mechanism by which prenatal immune activation can mediate changes in brain development and functions, even across generations. This review discusses the potential epigenetic mechanisms underlying the effects of prenatal infections, thereby highlighting how infection-mediated epigenetic reprogramming may contribute to the transgenerational transmission of pathological traits. The identification of epigenetic and transgenerational mechanisms in infection-mediated neurodevelopmental disorders appears relevant to brain disorders independently of existing diagnostic classifications and may help identifying complex patterns of transgenerational disease transmission beyond genetic inheritance. The consideration of ancestral infectious histories may be of great clinical interest and may be pivotal for developing new preventive treatment strategies against infection-mediated neurodevelopmental disorders.

## Introduction

Disruption of normal brain development is implicated in a number of neuropsychiatric diseases with neurodevelopmental origins, including schizophrenia, autism spectrum disorder and bipolar disorder.^1^ One commonality between these disorders is that the underlying pathological processes likely start early during fetal development and continues during subsequent brain maturation.^2^ The disruption of neurodevelopmental and maturational processes can lead to widespread and long-lasting abnormalities in central nervous system structure and functions. Typically, neurodevelopmental disorders are characterized by a heterogeneous group of symptoms affecting cognitive functions, emotions and behavior, thereby undermining basic human processes of perception and judgment. In view of their chronicity and severity, neurodevelopmental disorders impose tremendous burdens on the affected individuals, and in turn, on their families and society in general.^3^

Besides genetic contributions,^4^ various environmental factors appear to increase the risk for neurodevelopmental disorders.^1, 5^ Many of these factors operate during critical periods of central nervous system development. One of these factors is prenatal exposure to infection, which is increasingly recognized to play an important etiological role in various brain disorders with neurodevelopmental components, including schizophrenia,^5, 6^ autism spectrum disorder^7, 8^ and bipolar disorder.^6, 9^ Hence, prenatal exposure to immune challenges may be best viewed as a general vulnerability factor for neurodevelopmental brain disorders rather than a disease-specific risk factor.^10, 11^ Indeed, the adverse effects induced by prenatal infection may reflect an early entry into a deviant neurodevelopmental route, but the specificity of subsequent disease or symptoms is likely to be influenced by the genetic and environmental context in which the prenatal infectious process occurs. This notion is consistent with the emerging evidence suggesting that seemingly remote disorders, such as schizophrenia, autism spectrum disorder and attention-deficit/hyperactivity disorder share considerable amounts of risk factors and brain dysfunctions.^10, 11^ The presence of shared genetic and environmental risks among these illnesses has led to the proposal that they might lie along a continuum of genetically and environmentally induced neurodevelopmental causalities,^1, 12^ wherein prenatal infection may be one of the many factors that shape the eventual pathological outcomes.

The epidemiological link between prenatal infection and increased risk of neurodevelopmental brain disorders also receives strong support from experimental work in animal models.^13^ On the basis of human epidemiological findings, a number of translational rodent models have been established to explore the consequences of prenatal immune activation on brain and behavioral development. These models are based on maternal gestational exposure to specific infectious agents such as influenza virus or immune activating agents such as the bacterial endotoxin lipopolysaccharide or the viral mimic poly(I:C) (=polyriboinosinic–polyribocytidylic acid). Converging evidence from these models suggests that prenatal immune activation can negatively affect early fetal brain development and change the offspring’s neurodevelopmental trajectories, which in turn can lead to the emergence of behavioral and cognitive disturbances in later life.^13, 14, 15, 16^ The long-term behavioral and cognitive abnormalities induced by prenatal immune challenges in rodent models have already been extensively reviewed elsewhere (for review see Meyer,^13^ Meyer and Feldon,^15^ and Estes and McAllister^17^) and have recently been extended to models using primates with more advanced cortical development and functions.^18, 19, 20^

Despite the advances in this research field, the mechanisms whereby prenatal infection leads to long-term effects on central nervous system functions are only partially understood. In keeping with the crucial role of epigenetic remodeling in early development, it has recently been postulated that epigenetic modifications may be a critical molecular mechanism by which prenatal immune activation can mediate changes in brain development and functions.^21, 22^ The present review summarizes and integrates novel findings supporting this hypothesis and highlights how infection-mediated epigenetic reprogramming could be a molecular mechanism shaping disease risk across generations.

## Pathophysiological processes induced by maternal infection

**Figure 1:**
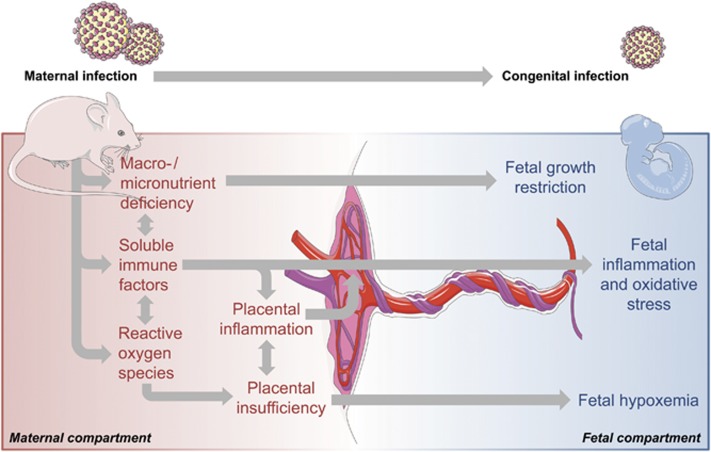
Post-acute pathophysiological effects of maternal infection during pregnancy. Some infectious pathogens (for example, rubella virus, cytomegalovirus, herpes simplex virus-2 and *Toxoplasma gondii*) can be vertically transmitted and lead to congenital infections, which in turn can result in severe developmental deficits. Maternal infection during pregnancy can further induce a number of pathophysiological responses, even if the pathogen is not vertically transmitted. These responses include the production of soluble immune factors such as cytokines and other mediators of inflammation, as well as reactive oxygen species. Some of these factors might cross the placental barrier and enter the fetal environment, thereby causing fetal inflammation and oxidative stress. Maternal infection during pregnancy can further induce inflammatory response in the placenta and cause placental insufficiency, which in turn can cause fetal hypoxemia. In addition, infection can cause (temporary) states of macronutrient and micronutrient deficiency, which limits the fetal availability of essential nutrients necessary for normal fetal development and growth. Finally, maternal infection during pregnancy can modify the microbial composition of the placenta, which might alter the development of the offspring’s microbiome (not shown).

Infection during pregnancy induces a spectrum of pathophysiological changes in the maternal host and fetal compartments.^23^ Figure 1 provides a schematic summary of the most relevant pathophysiological processes associated with maternal infection during pregnancy. The nature of these processes, and the resulting neurodevelopmental sequelae in the offspring, are likely influenced by a number of factors, including virulence of the pathogen, timing of infection and (genetic) susceptibility of the host. Interestingly, increased risk for neurodevelopmental disorders is observed following prenatal exposure to a variety of pathogens, including influenza, rubella, measles, polio and others (for review see Brown and Derkits,^5^ and Silasi *et al.*^23^). Only some of these pathogens are vertically transmitted and directly invade the fetal system. One prominent example showing transplacental transmission is rubella, which has long been known to be responsible for a spectrum of developmental abnormalities referred to as the congenital rubella syndrome.^24^ The majority of pathogens, however, stay refined to the maternal host upon infection during pregnancy.^23^ Even when lacking vertical transmission, maternal infection during pregnancy can induce long-term effects on the offspring, suggesting that the association between prenatal infection and neurodevelopmental disorders is, to a large extent, driven by factors that are common to all infections. In support of this notion, rodent studies provide substantial evidence for the hypothesis that inflammatory cytokines are one of the key players in re-wiring ongoing developmental processes into pathological trajectories following prenatal infection.^25, 26, 27^ Cytokines are increased during the acute phase response to infection and are important mediators of the host’s defense against infectious agents.^28, 29^ In the context of prenatal infection, some of the maternally produced cytokines can be transferred via the placenta, as shown in animals and human placental tissue,^30, 31^ and contribute to cytokine imbalances in the fetal system. In addition, the placenta and fetal organs can mount increased cytokine production in response to maternal infection.^32, 33, 34^

The post-acute effects of maternal infections are not limited to cytokine-associated inflammatory responses but further involve other pathophysiological processes, including nutrient deficiency and oxidative stress (Figure 1). Oxidative stress refers to an increase in production and/or decrease in elimination of reactive oxygen species. Reactive oxygen species are released as part of an activation cascade of innate immune cells to kill invading pathogens^35^ and can exert cytotoxic effects. Similar to cytokines, reactive oxygen species levels typically rise in response to an infection, which in turn can trigger oxidative stress in the fetal environment and negatively affect fetal brain development, both in humans and in animal models.^36^

Reduced availability of macro- and micronutrients for the fetus is yet another important post-acute effect of maternal infection (Figure 1). The infected host usually displays signs of sickness behavior, which is characterized by fever, malaise and reduced water and food intake.^37^ Reduction in food intake might indeed be of great relevance in the context of prenatal infection, with several studies highlighting a significant link between prenatal malnutrition and pathological brain development.^38, 39^ Studies in rats and mice suggest that the association between prenatal malnutrition and neurodevelopmental disorders might result, at least in part, from a reduction in the availability of micronutrients such as zinc and iron, which are crucial for normal brain development.^40, 41^

Finally, it should be noted that infection-induced pathophysiological changes in the placenta might amplify the response to maternal infection and the downstream pathological effects on the fetus. The placenta plays a key role in maternal–fetal interactions and expresses a variety of factors important for the homeostatic response to infectious agents.^42^ Maternal infections also typically disrupt the circulation and architecture of the placenta,^27, 43, 44^ as shown by studies in rodents and humans, which in turn can induce placental insufficiency and reduce the delivery of oxygen and essential nutrients to the developing offspring (Figure 1).

In summary, infection during pregnancy induces a number of post-acute pathophysiological processes in the maternal host, which in turn can all contribute to the disruption of normal brain development. As discussed in detail below, novel findings suggest that epigenetic modifications may represent an important molecular mechanism whereby these post-acute pathophysiological processes are translated into long-term brain abnormalities, even across generations.

## A novel role of epigenetic modifications in infection-mediated neurodevelopmental disorders

### A short overview of epigenetic mechanisms

**Figure 2:**
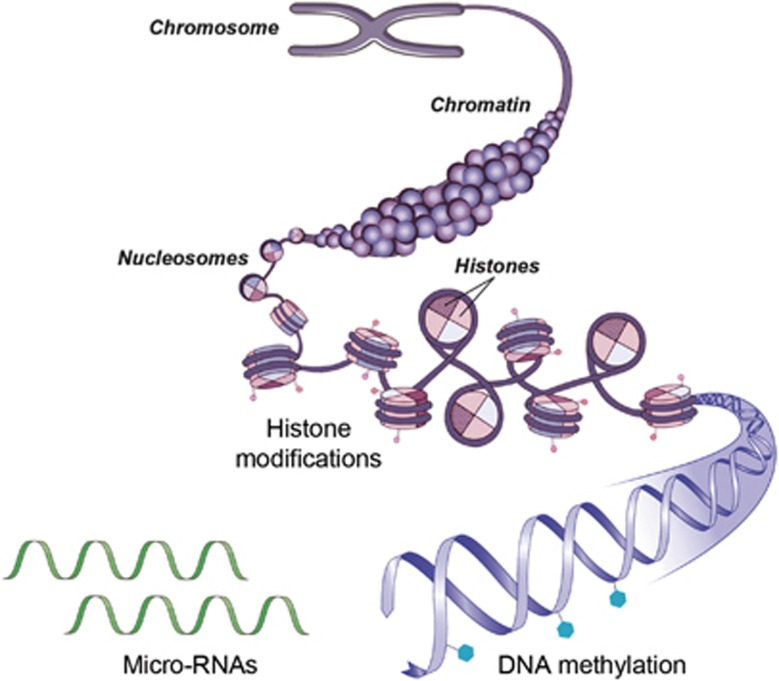
Schematic representation of major epigenetic mechanisms. The DNA–protein complex is referred to as chromatin. The functional unit of the chromatin is the nucleosome, which is composed of DNA wrapped around a core octamer of histone proteins. The DNA–histone interaction occurs at the N-terminal tails of these histones, which face outward and are sites for epigenetic marking known as histone modifications. These modifications represent a first major epigenetic mechanism modulating gene expression and involve methylation, phosphorylation, acetylation, ubiquitylation and sumoylation. DNA methylations at cytosine rings, typically found at CpG dinucleotides, are another important epigenetic mark that can influence gene expression. Finally, micro-RNAs (miRNAs), a class of small, non-coding RNAs, can control target gene expression post-transcriptionally. CpG, cytosine–phosphodiester–guanine.

‘Epigenetics’ refers to the combination of mechanisms that confer long-term and heritable changes in gene expression without altering the DNA sequence itself.^21^ Epigenetic programming is dynamic and responsive to different environmental exposures during development and includes several interrelated processes (Figure 2), including chromatin remodeling, histone modifications, DNA methylation and expression of microRNAs (miRNAs).^21, 22, 45^ Histone modifications are covalent post-translational modifications of histone proteins, which include, among others, methylation, phosphorylation, acetylation, ubiquitylation and sumoylation.^21, 22, 45^ Histone modifications can define the extent to which DNA is wrapped around the nucleosome core, thereby influencing the accessibility of the gene transcription machinery and subsequent gene expression. DNA methylation, on the other hand, consists of covalent methylation of cytosine rings that are found at cytosine–phosphodiester–guanine (CpG) dinucleotides.^21, 22, 45^ When located in distinct genomic regions such as gene promoter or enhancer sites, DNA methylation typically acts to repress gene transcription.^21, 22, 45^ Silencing of gene transcription by DNA methylation can be mediated by direct interference with the binding of transcription factors or enhancers to recognition elements that contain CpG dinucleotides, or through recruitment of methylated DNA-binding factors that in turn attract chromatin-inactivating complexes including histone deacetylases and histone methyltransferases. Finally, miRNAs are a class of small non-encoding RNAs (~22 nucleotides long) that can control target gene expression post-transcriptionally.^21, 22, 45^ Interestingly, recent studies have demonstrated that epigenetic mechanisms, such as DNA methylation, not only regulate the transcription of protein-encoding genes, but also the expression of miRNAs.^46^ Conversely, miRNAs can control the expression of important epigenetic regulators, including DNA methyltransferases and histone deacetylases. Hence, there is a dynamic regulatory network between different epigenetic pathways, which altogether organize gene expression profiles through transcriptional or post-transcriptional mechanisms.^21, 22, 45, 46^

### How can epigenetic modifications influence brain development?

A growing number of studies highlight the importance of epigenetic mechanisms in normal brain development.^45^ Interestingly, transcriptional changes in the brain occur more frequently during prenatal life as compared to any other age in life.^47^ A temporally precise and specific regulation of gene expression thus seems indispensable for normal brain development. This regulatory process involves methylation-related epigenetic modifications, which allow a fine-tuning of fetal gene expression according to specific stages of brain development.^48, 49, 50^ A critical role for DNA methylation in early brain development is also supported by the dynamic expression of DNA methyltransferases during prenatal life,^51^ and by studies using transgenic mouse models showing neurodevelopmental deficits in mice with mutations in the methyl-CpG-binding protein 2 (Mecp2) gene.^52, 53^ Furthermore, mice lacking distinct DNA methyltransferases (DNMTs) in the forebrain have been found to display severe learning and memory deficits, as well as impaired synaptic plasticity.^54, 55^

In addition to epigenetic processes involving DNA methylation, several other epigenetic mechanisms appear to be critical for neuronal development and functions. For example, it has been shown that histone modifications can regulate the conversion of oligodendrocytes into neural stem cells.^56^ Furthermore, Fischer *et al.*^57^ demonstrated in a mouse model of neurodegenerative disorders that increasing histone acetylation could promote synaptogenesis and augment cognitive functions.

Accumulating evidence suggests that miRNAs may similarly play a role in central nervous system development. For example, it has been shown that miRNAs in specific neuronal or glial cell populations show a dynamic expression pattern during brain development.^58, 59^ Moreover, a recent study reported distinct temporal patterns of miRNA expression in the brain throughout gestation, and from early neonatal to adult life.^60^ The developmentally regulated pattern of miRNA expression is indicative of a functional role of these molecules in normal brain development. However, our understanding of how miRNA can influence neurodevelopmental processes is still in its infancy and warrants further investigations.

The importance of epigenetic mechanisms in brain development is also supported by a plethora of findings demonstrating epigenetic alterations in neurodevelopmental disease models that are based on exposure to environmental adversities in early life. As reviewed in detail elsewhere^21, 45, 61^ stable epigenetic modifications may represent an important mechanism by which exposures to early-life environmental adversities can induce pathological consequences, even across multiple generations. Transgenerational transmission of disease susceptibility or epigenetic modification has been observed following early-life exposure to various environmental adversities (see Table 1 for prenatal environmental adversities), including prenatal or neonatal stress,^62, 63, 64, 65, 66, 67, 68, 69^ prenatal malnutrition,^70, 71, 72, 73, 74, 75^ endocrine disruptors^76, 77, 78, 79, 80, 81^ and chronic psychostimulant or alcohol intake.^82, 83^ The phenomenon of non-genetic transgenerational transmission of behavioral traits has gained increasing recognition in view of its potential importance in the etiology and treatment of multifactorial disorders.^61, 84, 85^ As discussed in the subsequent sections, recent research now suggests that similar effects can be induced by prenatal exposure to infection.

## Epigenetic and transgenerational effects of prenatal infection

### Epigenetic modifications induced by prenatal infection

Several recent studies sought to examine the putative effects of prenatal infection on epigenetic processes. Using a rat model of poly(I:C)-induced maternal immune activation, Hollins *et al.*^86^ revealed that prenatally infected offspring exhibited significant differences in the expression of miRNA in the entorhinal cortex, a brain area implicated in neurodevelopmental disorders. Interestingly, a large subset of these miRNAs were clustered within the *Dlk1-Dio3*-imprinted domain on 6q32, which is associated with schizophrenia, and were predicted to regulate pathways involved in synaptic remodeling, learning and memory formation.^86^ Maternal immune activation by poly(I:C) treatment in mice has also been found to alter histone modifications in the offspring. More specifically, offspring of poly(I:C)-treated mice displayed changes in promoter-specific histone acetylation and corresponding transcriptional changes, the latter of which affected genes associated with neuronal development, synaptic transmission and immune signaling.^87^

Accumulating evidence suggest that prenatal infection can also cause stable changes in DNA methylation. For example, Basil *et al.*^88^ found that prenatal exposure to the viral mimic poly(I:C) caused global changes in the level of DNA methylation in the adolescent mouse brain, including Mecp2 promoter hypomethylation. A recent study by Labouesse *et al.*^89^ extended those findings by assessing correlations between DNA-related epigenetic modifications, expression levels of corresponding genes and behavioral deficits. The authors showed that prenatal viral-like immune activation by poly(I:C) in mice induced methylation-related promoter remodeling of GAD1 and GAD2 in the prefrontal cortex. GAD1 and GAD2 encode for two isoforms of the rate-limiting enzyme for γ-aminobutyric acid biosynthesis. It was shown that offspring born to immune-challenged mothers displayed GAD1 and GAD2 promoter hypermethylation and associated reductions in the expression of the corresponding mRNA transcripts (GAD67 and GAD65), which in turn correlated with deficits in social interaction and impairments in working memory.^89^ These findings suggest that methylation-related epigenetic modifications at presynaptic GABAergic systems may represent a mechanism whereby maternal infection during pregnancy can induce long-term behavioral impairments in the offspring.

Using the same mouse model of poly(I:C)-induced maternal immune activation, a recent study by Richetto *et al.*^90^ examined genome-wide DNA methylation differences at single-nucleotide resolution by capture array bisulfite sequencing in the adult prefrontal cortex. It was shown that offspring of immune-challenged mothers displayed hyper- and hypomethylated CpGs at numerous loci and at distinct genomic regions.^90^ The differences in methylation were again associated with transcriptional changes of the corresponding genes, suggesting that the infection-induced epigenetic modifications had a functional impact on gene expression.^90^

Taken together, these findings indicate that prenatal infection can cause lasting changes in the offspring’s epigenome. The available data thus far show that viral-like maternal immune activation in early/middle (between gestation day (GD) 9 and 12)^87, 88^ or in late (GD15 and beyond)^86, 89^ gestation causes such changes. Recent evidence suggests, however, that the timing of viral-like immune challenge critically determines the specificity of infection-mediated epigenetic modifications,^90^ since early and late gestational window clearly differ in terms of methylation-related epigenetic modifications they induce.

### Transgenerational effects of prenatal infection

**Figure 3:**
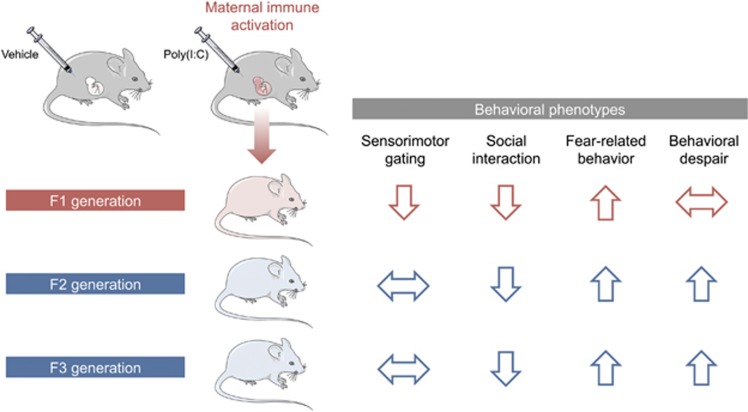
Summary of the transgenerational transmission and modification of behavioral deficits induced by prenatal immune activation. The use of a mouse model of viral-like immune activation, which was induced by maternal treatment with the viral mimetic poly(I:C), led to the recent discovery of transgenerational effects following prenatal immune activation (for details, see Weber-Stadlbauer *et al.*^92^). In this model, reduced sociability and increased fear-related behavior were similarly present in first-generation (F1) and second-generation (F2) offspring of immune-challenged ancestors. Sensorimotor gating impairments were confined to the direct descendants of infected mothers, whereas increased behavioral despair emerged as a novel phenotype in the second generation. The transgenerational effects were transmitted via the paternal lineage (not shown) and were stable until the third generation (F3), demonstrating transgenerational non-genetic inheritance of pathological traits following prenatal immune activation.

Recent studies extended the abovementioned findings by assessing whether behavioral and cognitive abnormalities emerging in the direct descendants (F1) of gestationally immune-challenged mothers could be transmitted across subsequent generations (F2 and F3) without any further immune exposures. Using the prenatal poly(I:C) administration model in mice, it was repeatedly shown that some of the behavioral abnormalities are not only present in the direct descendants of immune-challenged mothers, but are transmitted to the subsequent generations,^91, 92^ at least when the immune challenge was induced in early/middle pregnancy (that is, between GD9 and GD12). Interestingly, one study reported a transgenerational transmission of behavioral abnormalities mostly via the paternal lineage, which extended to the third (F3) generation of offspring (Figure 3).^92^ The paternal mode of transmission is consistent with other models of early-life adversities, such as pre- and neonatal stress and prenatal malnutrition.^62, 63, 64, 65, 66, 70, 71^ The fact that prenatal immune activation can cause transgenerational transmission of pathological phenotypes via the paternal lineage strongly suggests the involvement of epigenetic modifications in male gametes, which in turn could mediate epigenetic inheritance across generations.^21, 45, 63, 66^ Prenatal immune activation thus likely alters epigenetic marks in the germ line of the direct offspring, which resists erasure and epigenetic reestablishment during germ cell development.^21^

It remains to be determined further, however, why early-life adversities such as maternal infection largely spare the female germ cells. One possible explanation may relate to the differential developmental dynamics of male and female gametes. Germ cells start developing shortly after fertilization and rapidly proliferate until they migrate to the genital ridge, where sex determination occurs.^21^ Once the primordial germ cells are sexually differentiated, oocytes and male germ cells have different developmental dynamics; following sexual differentiation, the oocyte enters into meiosis and arrests until puberty, while male germ cells go into arrest until birth, when they undergo a phase of proliferation and then complete meiosis during puberty.^93^

In addition to its transgenerational effects on behavior, prenatal exposure to viral-like immune activation in mice was also found to modify transcriptional activity across generations.^92^ Using next-generation mRNA sequencing, it was shown that prenatal poly(I:C)-induced immune activation caused widespread gene expression changes in the brains of both the F1 and F2 generations. Intriguingly, while some transcriptional changes were uniquely present in either F1 or F2 offspring, others were common to both generations. Hence, the prenatal infection-induced transgenerational transmission of behavioral abnormalities (Figure 3) is associated with, and perhaps even mediated by, transgenerational modifications of gene expression.

Depending on the timing of the environmental insults or genetic loci, some of the epigenetic marks may affect only the germ line while sparing somatic tissues in the first generation. This may explain why certain behavioral deficits emerge as a novel phenotype only in the second and third generation of infected ancestors (Figure 3). Future studies are warranted in order to compare infection-induced epigenetic modifications in somatic cells and gametes. Such studies will help to further elucidate the mechanisms underlying the transgenerational modification and inheritance of brain pathology following prenatal immune activation.

## Potential mechanisms through which prenatal infection might induce epigenetic changes (across generations)

### The potential role of cytokines

As mentioned above, epidemiologic studies suggest that the risk of neurodevelopmental disorders is increased after prenatal exposure to a variety of viral, bacterial or protozoan pathogens, including those that are not vertically transmitted to the fetus. Based on these findings, it has been proposed that cytokine-associated inflammatory processes may be key in mediating the negative effects of maternal infection.^94^ Molecular work in mouse models of maternal immune activation has provided additional support for this hypothesis. For example, prenatal administration with specific cytokines such as interleukin-6 (IL-6) or IL-17a can mimic the long-term consequences of prenatal viral-like immune activation in mice.^25, 26^ Conversely, elimination of these cytokines from the maternal immune response by genetic interventions or blocking antibodies attenuates the long-term effects of prenatal immune challenge on brain and behavior.^25, 26^

Given these findings, it appears plausible that enhanced expression of inflammatory cytokines may be one of the factors that can directly alter epigenetic programs in the event of prenatal infection. In support of this notion, a study by Hodge *et al.*^95^ demonstrates that IL-6 increases the expression and biological activity of DNMT1. Some of the changes in DNMT1 activity may result from phosphorylation of the enzyme following exposure to IL-6, and the subsequent activation of downstream signaling pathways such as the AKT pathway.^95^ While most of the initial findings linking cytokines to epigenetic effects stem from experiments with cancer cell lines, it is likely that IL-6 regulates DNMT1 in several distinct cell types, including neurons and other brain cells. It was shown that IL-6 and DNMT1 can also influence cell migration,^96^ a process which is also essential in neurodevelopment. Recent studies also identified a direct influence of IL-17a on the epigenetic machinery through the modulation of histone deacetylases (HDACs). More specifically, IL-17a was shown to inhibit HDAC activity via the phosphoinositide-3-kinase (PI3K) pathway.^97^ Finally, one study found that TNF-alpha increases histone acetylation activity,^98^ suggesting that this inflammatory cytokine can act on the epigenetic machinery as well.

Exposure to prenatal inflammation stimuli has also been shown to cause Mecp2 promoter hypomethylation.^88^ Mecp2 is important for epigenetic regulation due to its ability to bind to methylated DNA and to chromatin modifying complexes such as HDACs. Therefore, Mecp2 is considered both an epigenetic ‘reader’ and a ‘writer’ (for review see Ausio^99^). Additional research is required, however, to examine more directly whether the effects of prenatal immune activation on Mecp2 promoter hypomethylation involves specific cytokines or other pathophysiological processes associated with inflammation.

Taken together, there is initial evidence supporting a role of cytokines in modulating the epigenetic machinery in the event of prenatal infection. Hence, increased fetal expression of inflammatory cytokines may be one of the upstream factors causing epigenetic reprogramming and associated neurodevelopmental changes in offspring of gestationally infected mothers.

### The potential role of folic acid

As previously discussed, maternal infection typically leads to temporary reduced food consumption, thereby reducing nutrient availability for the developing organism (Figure 1). Besides its effects on other micronutrients such as zinc and iron, reduced intake of micronutrients may lead to a transient deficiency of folic acid. Folic acid was shown to play a key role in the modulation of inflammatory responses: supplementation of patients with folic acid inhibits pro-inflammatory responses, such as inhibiting chemokine secretion and reducing the elevated levels of reactive oxygen species.^100^ Vice versa, mouse monocytes, under conditions of folate deficiency, show a 2- to 3-fold increase in the expression of several inflammatory cytokines, such as IL1b, IL-6 and TNFα.^101^ Folic acid has also long been implicated in neurodevelopmental processes, as exemplified by the presence of *spina bifida* in offspring of mothers who were deprived from folic acid during pregnancy.^102^ Moreover, imbalances in folate levels during pregnancy represent an important, epidemiologically valid risk factor for later psychiatric disease.^103^ Besides its established etiological role in neurodevelopmental disorders,^104^ low levels of folate are also present in patients diagnosed with schizophrenia.^105^ There is a growing body of evidence linking folic acid deficiency with autism as well.^106, 107, 108^ Interestingly, maternal folic acid supplementation has been associated with beneficial effects on neurodevelopment and autism.^103^ Moreover, mothers of children with autism have a significantly lower mean folic acid intake during early pregnancy compared to mothers of children with autism spectrum disorder.^107^

Representing one of the major dietary sources for methyl groups,^109^ folic acid is likely to have a direct influence on epigenetic processes during normal prenatal development. Indeed, a recent study identified a striking correlation between maternal folic acid and methylation status of a variety of genes in the offspring, some of which are related to neurological functions and to embryonic development.^110^ In the pathological context of maternal infection, decreased availability of folic acid may affect methylation-related epigenetic processes in the fetus, thereby contributing to infection-mediated epigenetic programming. Even though biologically plausible, however, this hypothesis still awaits direct examination in future studies.

### The potential role of the microbiome

The recent years have witnessed an increasing interest in the role of the microbiome in health and disease. The human body is composed of a complex ecosystem of microbial cells, approximating the same number as the number of human cells in the human body.^111^ There is a clear symbiotic link between host and microbe, wherein the microbiota is also key mediating for gene–environment interactions relevant to various physiological processes.^112^ Whereas initial studies mainly focused on obesity-related changes in the microbiome and its contribution to the disease phenotype, it has recently been shown that the microbiome can also influence behavior and contribute to brain development and function (for review see Cryan and Dinan^113^). While the fetal environment was initially thought to be entirely sterile, recent evidence suggests that some bacteria are present in the amniotic fluid^114^ and the placenta.^115^ One implication of these new discoveries is that the microbial composition of the developing offspring may be sensitive to environmental changes even during prenatal stages of life. In support of this notion, a recent study in mice demonstrated that prenatal viral-like immune activation alters the offspring’s composition of the gut microbiota.^116^ Notably, some of the main metabolic products of the gut microbiota are short-chain fatty acids, including sodium butyrate. Sodium butyrate can readily cross the blood–brain barrier and has been shown to modulate the expression of various genes in the prefrontal cortex, particularly those involved in neuronal excitation or inhibition.^117^ At the same time, sodium butyrate can interact with the epigenetic machinery by inhibiting HDACs.^118^ Therefore, aside from being a bacterial metabolite and a major source of energy for intestinal epithelial cells, sodium butyrate represents an efficient epigenetic regulator. Future studies are warranted in order to investigate whether infection-induced changes in gut microbiota may induce downstream changes in sodium butyrate, which in turn may affect the epigenetic machinery. Such changes would possibly also affect the fetus’ germ cells, and thereby, they could also contribute to the transgenerational transmission of behavioral traits induced by maternal immune activation.

## The influence of the precise timing of maternal infection

An important aspect that warrants further investigation relates to the potential influence of the precise timing of prenatal immune activation, both in terms of its effects on the offspring’s epigenome and on the transgenerational transmission of pathological traits. Exposure to environmental insults during distinct gestational stages likely affects different neurodevelopmental events, developing brain regions, and cellular maturational processes, which in turn might shape the specificity of long-term brain dysfunctions in the offspring.^34^ In support of this notion, numerous rodent studies highlighted a critical influence of the precise timing of prenatal infection with regards to its long-term brain and behavior consequences in the offspring.^119, 120^

It has long been recognized that epigenetic processes follow timed developmental patterns during embryogenesis and fetal development.^121, 122^ It thus follows that maternal immune activation induces differential epigenetic modifications in the offspring depending on the precise timing of exposure. Indeed, a recent mouse study provided the first line of evidence suggesting that the epigenetic effects of prenatal immune activation are critically influenced by the timing of prenatal immune challenge.^90^ While immune activation in early and late gestation affected a relatively small number of common genes, the two prenatal conditions mostly showed methylation differences in distinct loci.^90^ Moreover, the differential epigenetic effects of early and late prenatal immune activation were associated with distinct transcriptional changes and behavioral abnormalities.^90^ Against this background, it appears essential to further explore whether and how timing-dependent effects are also reflected by the nature of the transgenerational effects induced by prenatal infection.

## Conclusions

The investigation of possible immune mechanisms in neurodevelopmental disorders is a long-standing area of research that continuously attracts the attention from basic scientists and clinicians alike. Within this neuroimmune framework of brain disease, a great deal of interest has been centered on the possible contribution of infections in prenatal life. The recent outbreak of Zika virus and its accompanying risk for severe brain anomalies such as microcephaly^123^ has moved the risks of prenatal infection in the spotlight of public attention.

Modeling the consequences of prenatal infection in animals has led to the new discoveries that this early-life adversity induces stable epigenetic modifications in the offspring. There is increasing recognition of and evidence for altered epigenetic programming in the pathogenesis of neurodevelopmental disorders, including schizophrenia, autism and bipolar disorder.^124, 125, 126, 127^ Against these backgrounds, the novel findings of infection-mediated epigenetic modifications raise the intriguing possibility that prenatal exposure to immune challenges may be one of the environmental factors causing epigenetic abnormalities in these disabling brain disorders. Since at least some of these epigenetic modifications may be modifiable throughout life,^22, 128^ these findings may help to open new avenues for symptomatic or even preventive treatments in subjects with prenatal infectious histories.

The transgenerational transmission and modification of brain pathology following prenatal immune activation highlights a novel pathological aspect of this early-life adversity in shaping disease risk across generations. Given that prenatal infection is implicated in a variety of neurodevelopmental brain disorders, these new findings appear relevant to brain disorders independently of existing diagnostic classifications and may help identifying complex patterns of transgenerational disease transmission beyond genetic inheritance. These discoveries may further emphasize the importance for global health strategies in focusing on the development and promoting vaccinations, in particular for women who are pregnant or those who plan to become pregnant. Indeed, vaccinations may have the potential to prevent a wide spectrum of neuropsychiatric and neurological disorders associated with prenatal infection, and they may even prevent possible pathological effects across generations. Unfortunately, the limited access to vaccines in certain countries, along with the continuous trend against vaccinations within certain groups of the society, have led to a resurgence of serious infectious diseases, including measles, rubella and polio.^129^ Hence, there are obvious challenges for future attempts to implement vaccinations as preventive means against infection-mediated neurodevelopmental disorders. The continuous implementation and integration of preclinical studies and human epidemiological research will be pivotal in overcoming these hurdles and may eventually help reducing or even preventing the burdens associated with infection-mediated neurodevelopmental disorders.

## Figures and Tables

**Table 1:** Main manuscripts that investigated the association between prenatal environmental adversities, epigenetic changes and/or transgenerational effects

*Prenatal environmental adversity*	*Species*	*Epigenetic alterations*	*Trans-generational effects*	*References*
*Endocrine disruptor*				
Vinclozolin	Rat Mouse	DNA methylation DNA methylation	Yes Yes	Anway *et al.*,^76^ Skinner *et al.*^77^ Stouder *et al.*,^78^ Guerrero-Bosagna *et al.*^81^
Bisphenol A	Mouse	DNA methylation	Yes	Faulk *et al.*,^79^ Wolstenholme *et al.*^80^

High-fat diet	Mouse	Histone modifications	Yes	Dunn and Bale,^71^ Masuyama *et al.*^75^

Stress exposure	Mouse Rats	miRNA expression patterns miRNA expression patterns, DNA methylation	Yes Yes	Morgan and Bale^67^ Yao *et al.*,^68^ Jensen-Pena *et al.*^69^

Malnutrition	Humans Rats	DNA methylation Histone modifications	Yes	Veenendaal *et al.*,^70^ Heijmans *et al.*^73^ Zheng *et al.*^74^

Immune activation	Rats Mouse	miRNA expression patterns Histone modifications, DNA methylation	Yes	Hollins *et al.*^86^ Tang *et al.*,^87^ Basil *et al.*,^88^ Labouesse *et al.*,^89^ Richetto *et al.*,^90^ Ronovsky *et al.*,^91^ Weber-Stadlbauer *et al.*^92^
